# Moral Observer-Licensing in Cyberspace

**DOI:** 10.3390/bs12050148

**Published:** 2022-05-17

**Authors:** Yawei Ran, Yubo Hou, Zhiwen Dong, Qi Wang

**Affiliations:** 1 School of Psychological and Cognitive Sciences and Beijing Key Laboratory of Behavior and Mental Health, Peking University, Beijing 100871, China; ranyawei@126.com (Y.R.); 2001110672@stu.pku.edu.cn (Z.D.); 2 Culture & Cognition Laboratory, College of Human Ecology, Cornell University, Ithaca, NY 14853, USA

**Keywords:** moral observer-licensing, immoral behavior, role involvement, moral credits model, internet, social media

## Abstract

Moral observer-licensing happens when observers condone actors’ morally questionable conduct due to the actors’ history of moral behaviors. In four studies (N = 808), we investigated this phenomenon in the context of cyberspace and its contributing factors and boundary conditions. The pilot study determined what participants perceived as typically moral and immoral behaviors in cyberspace. Then, in Study 1, participants condemned a story character’s online immoral behavior less often when they were informed of the character’s prior online moral behavior than when they were not, which indicates moral observer-licensing in cyberspace. Study 2 confirmed the presence of moral observer-licensing in cyberspace and further demonstrated that a character’s prior moral or immoral behavior online respectively reduces or intensifies the perceived negativity of the character’s subsequent immoral behavior. Finally, Study 3 showed that participants who identified with the victim in a hypothetical scenario showed less forgiveness and more condemnation of a character’s immoral behavior than those who identified with the perpetrator or the bystander. These findings are of theoretical and practical significance for our understanding of cyber ethics.

## 1. Introduction

Moral licensing is the phenomenon of people permitting immoral or problematic behaviors if the actor initially behaves in a moral way [1]. The most studied type of moral licensing is moral self-licensing, where individuals condone their own immoral behavior when they hold a good record of past moral behaviors [2,3,4,5,6,7,8]. However, research on moral observer-licensing, where observers condone actors’ morally questionable conducts due to the actors’ history of moral behaviors, is relatively scarce [1,9]. The present research examined factors influencing moral observer-licensing in the context of cyberspace, where behavior ethics have become an increasing concern in our digitally mediated society [10].

### 1.1. Moral Observer-Licensing and Theoretical Explanations

Moral observer-licensing has been observed in everyday judgment. For example, when evaluating immoral behaviors such as a hit-and-run or marital infidelity, people show less condemnation if they know that the actor has previously demonstrated ethical behaviors such as helping the homeless [1]. Similarly, subordinates are more tolerant of their supervisors’ abusive behaviors if the supervisors have acted morally before [11]. According to the moral credentials model, prior moral behaviors enable the actor to establish a moral image which then serves as credentials that reflect the moral resources accumulated by the actor, and thus prove that the actor is not an immoral person [12]. As a result, later immoral behaviors by the actor can be condoned, where observers choose to either deny the immoral quality of these behaviors or to perceive the actor from a positive perspective [1,6,9,13,14].

In a similar vein, the moral credits model assumes that everyone has an account in the moral bank consisting of moral deposits represented by moral behaviors, as well as moral debts brought by immoral behaviors [6,9,14,15,16,17]. Moral deposits and debts can cancel each other out to keep the account in balance. When moral debts are offset by moral deposits, immoral behaviors tend to be condoned. The moral credits model is an extension of Nisan’s moral balance theory [18,19], which argues that moral self-regard and moral equilibrium are influential factors in moral evaluation. Moral self-regard is a temporary state that varies under external influences, and it can be easily improved by moral behaviors and reduced by immoral ones [20]. In contrast, moral equilibrium is stable and determines the threshold of moral evaluations. Moral self-regard always fluctuates around moral equilibrium: when moral behaviors raise moral self-regard to a level higher than moral equilibrium, immoral behaviors are condoned, whereas when moral self-regard drops below moral equilibrium, a greater amount of moral behavior is expected in order to increase moral self-regard.

Although both the moral credentials model and the moral credits model could explain moral observer-licensing, there are important differences between the two theories [21,22]. According to the moral credentials model, once the actor establishes a moral image, his or her later moral transgressions are cast in a positive light and reconstrued as not immoral. That is, a good person does everything right. In contrast, according to the moral credits model, following the actor’s moral behaviors, his or her subsequent transgressions are still viewed as immoral, although the transgressions can be condoned to some degree as they are offset by the actor’s moral history. That is, a good person also makes mistakes. As illustrated by the metaphor that Miller and Effron [1] (p. 126) used to distinguish the two theoretical models, “If moral credits function like currency that can be used to ‘‘purchase’’ a license to commit immoral behavior, moral credentials function like a character witness on which one can call to testify that subsequent behavior is not immoral.” It is theoretically important to further distinguish the two models in empirical research. 

### 1.2. Role Involvement and Moral Licensing

Although observers may give license to immoral behaviors based on the actor’s prior moral behaviors, differences may exist across situations [23,24]. When an immoral behavior occurs, individuals may play different roles in the incident as the perpetrator, the victim, or the bystander. Depending on what role they play, individuals judge the incident from different perspectives and then give different responses [25,26,27]. For example, after an unfair event, the perpetrators are more likely to show guilt, the victims are more likely to show anger, and the bystanders are more likely to show moral indignation [26]. When evaluating aggressive behaviors, the victims often find the behaviors to be more unreasonable and invasive than do the perpetrators, who tend to justify and downplay the harm done [28,29,30,31].

As observers of an unfair event, individuals may identify themselves with the perpetrator, the victim, or the bystander. This role involvement can, in turn, affect observers’ responses and moral evaluations [1]. Research has shown that individuals tend to perceive themselves and others in terms of particular social categories and further determine which groups they belong to [32,33,34]. By establishing identification with in-groups, individuals uphold values common in their groups. Moreover, they gradually perceive in themselves more in-group characteristics and less or no out-group characteristics [35], which can further lead to in-group preference and out-group bias [23,36,37]. Thus, by identifying with the perpetrator, the victim, or the bystander, observers may make moral judgement in line with their identified roles. The effect of role involvement and identification in moral observer-licensing has yet to be examined.

### 1.3. The Current Research

With the vast popularity of the Internet and social media, moral observer-licensing may become increasingly common in cyberspace. Although cyberspace is often characterized by virtuality and anonymity [38,39,40], it can be seen as an extension of the real world, and thus it encompasses patterns of behavior common in real life [41,42,43,44]. Indeed, some patterns of behavior, including moral observer-licensing, may be even more salient in cyberspace because of its virtuality and anonymity. Individuals may feel freer in this context to express themselves without worrying about others’ reactions compared with in-person interactions [10,39,45]. That is, the concerns for the consequences of one’s own actions and the social constraints on one’s actions and judgments tend to be reduced in cyberspace [46]. 

We conducted four studies to examine moral observer-licensing in the context of cyberspace. The pilot study used a survey to determine what participants typically perceived as moral behaviors and immoral behaviors in cyberspace. Based on the findings of the pilot study, hypothetical scenarios were created in Study 1 in which participants were asked to judge a character’s online immoral behavior when they were informed (moral behavioral history condition) or not informed (no behavioral history condition) of the character’s prior online moral behavior. We hypothesized that participants in the moral behavioral history condition would have less condemnation for the character’s immoral behavior than those in the no behavioral history condition, thus showing moral observer-licensing in cyberspace. Study 2 replicated the Study 1 findings and further examined the mechanism underlying moral observer-licensing in cyberspace in light of the moral credentials model and the moral credits model. Participants judged the online immoral behavior of a story character who had a moral history (moral behavioral history condition), an immoral history (immoral behavioral history condition), or no history (no behavioral history condition). We hypothesized that moral observer-licensing in cyberspace would be better explained by the moral credits model, whereby online immoral behaviors would be condoned if there were prior online moral behaviors, although they would still be judged as immoral. Finally, Study 3 examined whether moral observer-licensing in cyberspace would be influenced by observers’ role involvement. Participants were presented with a hypothetical scenario that involved a character with or without a moral history, in which participants would share a group identity with the perpetrator, the victim, or the bystander. We hypothesized that participants who identified with the victim would show reduced moral observer-licensing than those who identified with the perpetrator or bystander. Taken together, we expected moral observer-licensing to be prevalent in cyberspace, associated with the actor’s prior good deeds, and influenced by observers’ role involvement.

#### Ethics Statement

This project was approved by the Institutional Review Board (IRB) for Human Participants of Peking University (Protocol #2015-03-03c). The participants provided informed written consent. All relevant data are within the manuscript and its Supplementary Materials.

## 2. Pilot Study

The aim of the pilot study was to establish typically moral and immoral behaviors in cyberspace, which would then be used as materials in subsequent studies. 

### 2.1. Method

#### 2.1.1. Participants

A total of 63 undergraduate students at Peking University, China, volunteered to participate (*mean age =* 23.32 years, *SD =* 3.36; 34 women, 29 men). Participants had used the Internet for 9.73 years on average (*SD* = 2.95), and they reported spending on average 30.03 h (*SD* = 18.91) per week online. The participants were from diverse academic disciplines and were recruited through the Bulletin Board System of Peking University.

#### 2.1.2. Procedure

To avoid idiosyncrasy and identify the most typical behaviors, we created a list of 16 immoral behaviors, 9 moral behaviors based on prior research [10,40,41,45], and general societal concerns regarding online behaviors (see Table 1). Participants were individually tested in the lab. They were asked to indicate which behaviors on the list were the most immoral and which were the most moral in cyberspace, and to choose 3 out of the 16 immoral behaviors and 2 out of the 9 moral behaviors. 

### 2.2. Results and Discussion

Among the 16 immoral behaviors, “Using or changing others’ accounts or personal profiles online without authorization” and “Internet fraud” (e.g., setting up a fake E-commerce platform) were the most frequently chosen ones. Among the moral behaviors, “Supporting social assistance and other charitable activities” was the most popular choice (see Table 1). The two immoral behaviors and one moral behavior most frequently chosen by participants were used as the experimental materials in the subsequent studies. 

## 3. Study 1

In Study 1, we aimed to examine whether the phenomenon of moral observer-licensing was present in cyberspace. As cyberspace is an important extension of the real world and plays an increasingly significant role in regulating individuals’ sense of self and social behavior [10,41,42,44], we expected to observe moral observer-licensing in this context.

### 3.1. Method

#### 3.1.1. Participants

A meta-analysis found that the estimated effect size of moral licensing was at *d* = 0.31 [47]. A power analysis (G*Power 3.1) [48] showed that a sample size of 330 would be needed to achieve a power of 0.8 to detect an effect with a size of *d* = 0.31 and *α* = 0.05 in an independent sample *t*-test. We recruited 355 college students (*mean age* = 21.26 years, *SD* = 1.75) at Peking University (113 women and 242 men) to participate in the study. The participants had used the Internet for an average of 9.46 years (*SD* = 3.09), and they spent, on average, 31.35 h (*SD* = 21.38) per week online. They each received an RMB of 10 for their participation. The participants were from diverse academic disciplines and were recruited through the Bulletin Board System of Peking University.

#### 3.1.2. Procedure

The participants were individually tested in the lab, and each received a survey to complete. They were randomly assigned to a moral behavioral history (n = 178) or to a no behavioral history condition (n = 177). In the moral behavioral history condition, the participants were presented with a moral behavior scenario about a character, Li, followed by a scenario of Li’s immoral behavior. For the participants in the no behavioral history condition, only the scenario of Li’s immoral behavior was presented. A gender-neutral name was chosen for the character to avoid any potential influence of gender on participants’ judgment. 

The moral behavior scenario was as follows:

“*Li supported a charitable activity online. As an experienced Internet surfer, Li launched several campaigns online for a charity to help people who suffered from the Wenchuan Earthquake. Because it was shared by many people on the Internet, the campaigns raised nearly RMB 800,000 in the end, which greatly contributed to the reconstruction of Wenchuan*.”

The immoral behavior scenario was as follows:

“*Li used others’ accounts online without authorization. To upgrade his/her individual level in online games, Li hacked into other players’ accounts and tampered their data. What’s worse, Li stole other players’ accounts in online games and gave them to his/her friends as gifts*.”

After reading the second scenario, the participants of both conditions were instructed to evaluate Li and his/her transgression in the scenario. Following prior research on moral observer-licensing, the Condemnation Scale [49] was used as the dependent measure. Participants were asked to rate on seven-point scales (from 1 = “not at all” to 7 = “very much”) how much they agreed with each of the nine statements (e.g., “How moral do you find the target?”—reverse scored; and “How much should the target be blamed?”, “How much did the target deserve to be punished?”, and “How excusable was the transgression?”- reverse scored). A mean score of condemnation was calculated across the nine items and submitted to analysis, with higher scores indicating greater condemnation. Note that the Condemnation Scale contains four items referring to the perpetrator and five items referring to the transgression. Separate analyses of the two types of items across Studies 1 through 3 yielded consistent patterns of results for moral observer-licensing. The results based on the entire scale were reported. The internal reliability of the scale was Cronbach’s α = 0.86 for this sample, which was consistent with prior research [49].

### 3.2. Results and Discussion

**Figure 1:**
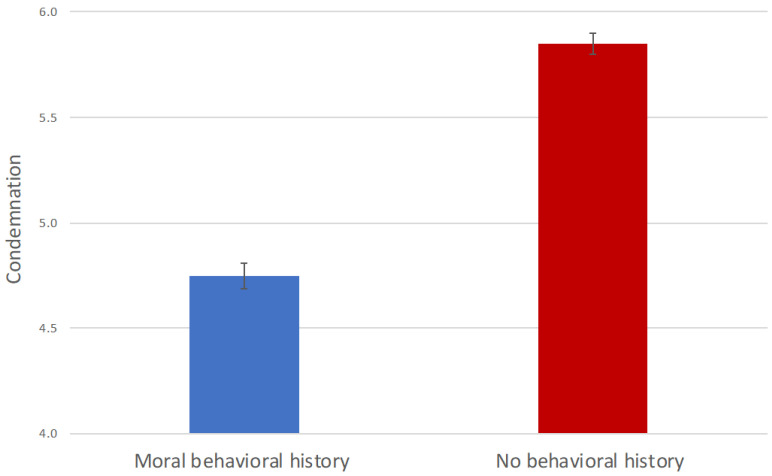
Condemnation of immoral behavior by condition. The error bars represent the standard errors of the means. The data are shown from the neutral point four of the Condemnation Scale.

Inspection of the data showed that the condemnation score was approximately normally distributed within each condition. An independent sample t-test on the condemnation score showed that participants in the moral behavioral history condition (*M* = 4.75, *SD* = 0.86) scored lower than those in the no behavioral history condition (*M* = 5.85, *SD* = 0.68), *t*(353) = −13.25, *p* < 0.001, *d* = −1.41 (see Figure 1). In addition, one-sample *t*-tests showed that the condemnation scores of both groups differed significantly from the neutral point four of the seven-point scale of the condemnation rating with *t*(177) = 11.63, *p* < 0.001, *d* = 0.87 for the moral behavioral history condition and *t*(176) = 35.89, *p* < 0.001, *d* = 2.70 for the no behavioral history condition.

Thus, when the participants were informed of the actor’s previous online moral behavior, they showed less condemnation to the actor’s later online immoral behavior compared with those who were unaware of the prior moral behavior. The finding provides evidence for moral observer-licensing in cyberspace. Although the popularity of cyberspace has changed individuals’ social relations and interactive modes, moral judgment processes online appear to be consistent with those in the real life [10,50,51,52]. The change of reality to virtuality has not transferred the path of peoples’ moral lives from “authentic” to “virtual,” but it extends the moral cognitive model of observers to the digitalized space of the Internet.

Notably, although the participants in the experimental group exhibited moral observer-licensing, their average condemnation score was significantly greater than four (i.e., neutral), which indicates that they still recognized the immorality of the actor’s immoral behavior. This finding suggests that the moral credits model may better explain cyberspace moral observer-licensing in the current context. We examined this question further in Study 2. 

## 4. Study 2

In Study 2, we examined moral observer-licensing in cyberspace from the perspectives of the moral credentials model and the moral credits model. Built on the findings of Study 1, we expected that online immoral behaviors would be condoned if there were prior online moral behaviors. However, the online immoral behaviors would still be perceived as immoral according to the moral credits model, rather than being reconstrued as moral according to the moral credentials model [1,14,21,22]. In other words, we expected moral observer-licensing in cyberspace to be better explained by the moral credits model. In line with the moral credits model and the moral balance theory [1,18,19], we also included a condition in which online immoral behaviors would be further condemned if there was a prior immoral history.

### 4.1. Method

#### 4.1.1. Participants

A power analysis [48] showed that a sample size of 159 would be needed to achieve a power of 0.8 to detect an effect with a size of *f* = 0.25 and *α* = 0.05 in a one-way ANOVA. We recruited a total of 170 college students at Peking University to take part in this study (*mean age* = 21.38 years, *SD* = 2.32, 68 women and 102 men). The participants had, on average, used the Internet for 8.74 years (*SD* = 3.49), and they spent, on average, 29.84 h (*SD* = 19.90) per week online. They each received an RMB of 10 for their participation. The participants were from diverse academic disciplines and were recruited through the Bulletin Board System of Peking University.

#### 4.1.2. Procedure

The participants were individually tested in the lab, and each received a survey to complete. They were randomly assigned to one of the following three conditions:

Moral behavioral history condition (n = 56): this condition was identical to the moral behavioral history condition in Study 1 where the participants were presented with scenarios of a moral behavior and then an immoral behavior of the character, Li.

Immoral behavioral history condition (n = 57): the participants were presented with scenarios of two immoral behaviors of Li. The first immoral behavior was about Li paying people to praise her/his e-shop, and the second was about Li using others’ accounts online without authorization (i.e., the same immoral behavior presented in the moral behavioral history condition). 

The first immoral behavior scenario was as follows:

“*As the owner of an e-shop, Li would like to boost the reputation of his/her store which was very important to attract customers. Instead of asking customers for feedback, Li spent a lot of money to pay part-time workers, who were non-customers, to praise his/her e-shop online. Within a month, Li’s e-shop reached the highest level of reputation*.”

No behavioral history condition (n = 57): Only the immoral behavior was presented, where Li used others’ accounts online without authorization (i.e., the same immoral behavior presented in the two behavioral history conditions). 

The participants were instructed to evaluate Li’s immoral behavior on the Condemnation Scale [49]. As in Study 1, a mean score of condemnation was calculated across the nine items and submitted to analysis, with higher scores indicating greater condemnation. The internal reliability of the scale was Cronbach’s α = 0.82 for this sample.

### 4.2. Results and Discussion

**Figure 2:**
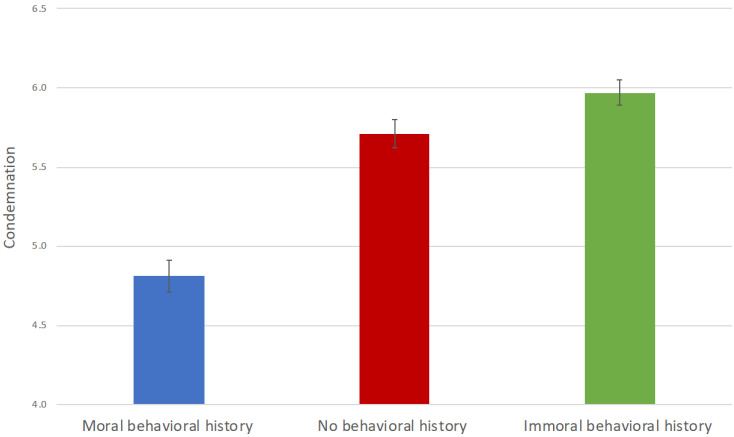
Condemnation of immoral behavior by condition. The error bars represent the standard errors of the means. The data are shown from the neutral point four of the Condemnation Scale.

Inspection of the data showed that the condemnation score was approximately normally distributed within each group. An analysis of variance (ANOVA) revealed a significant main effect of condition on the condemnation score, *F*(2, 167) = 46.15, *p* < 0.001, *η*^2^ = 0.36. Follow-up tests showed that the participants in the moral behavioral history condition (*M* = 4.81, *SD* = 0.73) had lower scores than those in the no behavioral history condition (*M* = 5.71, *SD* = 0.69), *t*(167) = −7.12, *p* < 0.001, *d* = −1.27, as well as those in the immoral behavioral history condition (*M* = 5.97, *SD* = 0.60), *t*(167) = −9.17, *p* < 0.001, *d* = −1.75 (see Figure 2). The participants in the immoral behavioral history condition and the no behavioral history condition did not significantly differ from each other. In addition, one-sample t-tests showed that the condemnation scores of all groups differed significantly from the neutral point four of the seven-point scale of the condemnation rating, with *t*(55) = 8.27, *p* < 0.001, *d* = 1.11 for the moral behavioral history condition, *t*(56) = 18.61, *p* < 0.001, *d* = 2.46 for the no behavioral history condition, and *t*(56) = 25.00, *p* < 0.001, *d* = 3.31 for the immoral behavioral history condition.

Thus, the participants who were informed of the moral history of the actor expressed the least condemnation for the actor’s online immoral behavior. This finding again confirms the presence of moral observer-licensing in cyberspace. Furthermore, across all three groups, the average condemnation scores were significantly greater than four (i.e., neutral), indicating that participants condemned the immoral behavior regardless of condition. This finding supports the moral credits model, which predicts that people do not deny the immorality of an actor’s immoral behavior even when they have less condemnation for the immoral behavior in light of the actor’s prior moral history [14,15,17]. The finding is not in line with the moral credentials model that predicts denial of the immorality of immoral behaviors during moral licensing [1]. In contrast to moral credits that offset or reduce the negative impact of a transgression, moral credentials make a morally questionable behavior appear as if it were not a transgression. Our findings suggest that in the current context, people give moral credits instead of credentials to others who conduct immoral deeds online following an established moral history. 

Furthermore, the participants who were informed of the immoral history of the actor appeared to express the most condemnation for the actor’s additional online immoral behavior, although their scores did not differ significantly from those in the no history group. It is possible that the seven-point scale limited the potential variability between the immoral history and no history conditions. Nevertheless, these findings lend additional support to the moral credits model and the moral balance theory [1,18,19]. When observers are made aware of an actor’s past moral behaviors, their moral evaluation of the actor is raised above the moral equilibrium and they tend to license the actor’s subsequent immoral behaviors. In contrast, when observers are made aware of the actor’s prior immoral behaviors, their moral evaluation of the actor is reduced to below the moral equilibrium and, in turn, they condemn any additional immoral behaviors. Our findings further suggest that the moral credit provided by a prior good deed might be greater than the moral debt brought about by a prior bad deed when compared with the control condition where the prior moral history was unknown. In other words, switching behaviors from good to bad might result in forgiveness to a greater degree than the amount of punishment from consecutive bad behaviors [53]. It would be interesting to verify this observation in future research.

## 5. Study 3

In Studies 1 and 2, the participants made moral judgments as observers. In many real-life situations, observers’ group memberships or role involvement may affect the extent to which they grant license to the actor based on a moral behavioral history [1]. We examined in Study 3 the effect of the observers’ role involvement on moral observer-licensing in cyberspace. Observers may be involved in different roles given their social identification and group membership, which may further lead to different judgements of immoral behavior. In line with prior research on in-group favoritism and out-group bias in social attribution and moral judgement [23,36,37], we expected moral observer-licensing to be less salient when participants shared a group identify with the victim than when they shared a group identity with the perpetrator or the bystander. In other words, when participants shared a group identity with the victim, they would have greater condemnation for an immoral behavior despite the actor’s prior moral history.

### 5.1. Method

#### 5.1.1. Participants

A power analysis [48] showed that a sample size of 158 would be needed to achieve a power of 0.8 to detect effects with a size of *f* = 0.25 and *α* = 0.05 in a 2 × 3 ANOVA. A total of 220 undergraduate students at Peking University were recruited to take part in this study (*mean age* = 20.77 years, *SD* = 2.55; 117 women and 103 men). The participants had, on average, used the Internet for 8.18 years (*SD* = 2.86) and spent, on average, 26.53 h (*SD* = 23.24) per week online. They received an RMB of 10 for their participation. The participants were from diverse academic disciplines and were recruited through the Bulletin Board System of Peking University.

#### 5.1.2. Procedure

The participants were individually tested in the lab, and each received a survey to complete. They were randomly assigned to one of the six conditions in a two (history: moral behavioral history vs. no behavioral history) × three (role: perpetrator, victim, or bystander) between-subjects design.

In the moral behavioral history condition (n = 37, 35, and 42, for the perpetrator, victim, and bystander conditions, respectively), a scenario of the character Li’s moral behavior (Li supported a charitable activity online) was first described, followed by a scenario of Li’s immoral behavior (Li used others’ online accounts without authorization). In the no behavioral history condition (n = 35, 34, and 37, for the perpetrator, victim, and bystander conditions, respectively), only the scenario of Li’s immoral behavior was presented. The moral and immoral behaviors were the same as those used in Study 1.

In the perpetrator condition, the participants were told that the character Li was a student at Peking University and no group identity information regarding the victim was provided. In the victim condition, the participants were told that the online account used by Li belonged to a student at Peking University and no group identity information regarding Li was provided. In the bystander condition, no group identity information was provided regarding either Li or the victim. 

The participants were then instructed to evaluate Li’s immoral behavior on the Condemnation Scale [49]. The internal reliability of the scale was Cronbach’s α = 0. 81 for this sample.

### 5.2. Results and Discussion

**Figure 3:**
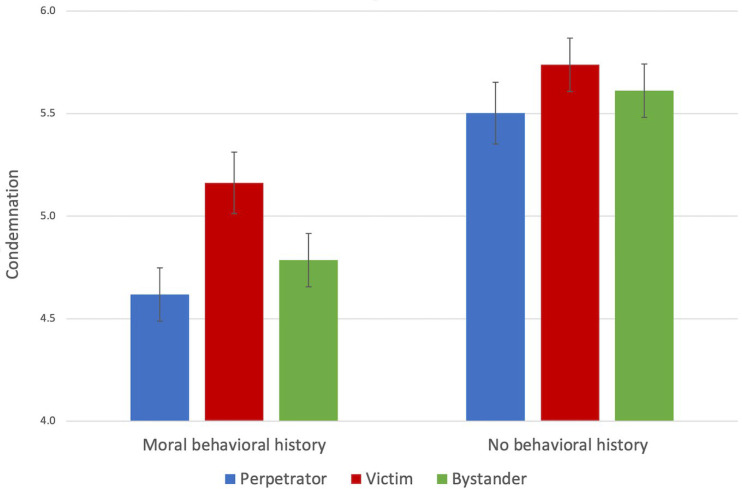
Condemnation of immoral behavior by condition. The error bars represent the standard errors of the means. The data are shown from the neutral point four of the Condemnation Scale.

An inspection of the data showed that the condemnation score was approximately normally distributed within each group. A 2 (history) × 3 (role) analysis of variance (ANOVA) on the condemnation score revealed a significant main effect of history, *F*(1, 214) = 46.39, *p* < 0.001, *η^2^* = 0.178. As predicted, the participants in the moral behavioral history condition (*M* = 4.85, *SD* = 0.87) condemned the actor’s immoral behavior less than those in the no behavioral history condition (*M* = 5.62, *SD* = 0.80). There was also a significant main effect of role involvement, *F*(2, 214) = 4.01, *p* = 0.020, *η^2^* = 0.036. Follow-up tests revealed that the participants in the victim condition (*M* = 5.45, *SD* = 0.87) condemned the actor’s immoral behavior more than those in the perpetrator condition (*M* = 5.05, *SD* = 0.95), *t*(139) = 2.85, *p* = 0.005, *d* = 0.44 and those in the bystander condition (*M* = 5.17, *SD* = 0.91), *t*(146) = 2.01, *p* = 0.046, *d* = 0.31. The difference between the perpetrator and the bystander conditions was not significant. There was no significant history–role interaction (see Figure 3). 

Consistent with Studies 1 and 2, when the participates were made aware of the actor’s previous moral behavior, their condemnation of the actor’s subsequent immoral online behavior was attenuated, regardless of their role involvement. This finding provides further evidence for moral observer-licensing in cyberspace. Furthermore, the participants’ role involvement influenced their degree of moral observer-licensing. When the participants shared the victim’s group identity and membership, their condemnation of the actor’s immoral online behavior was the harshest. In contrast, when participants shared the perpetrator (actor)’s group identity and membership, their condemnation of the actor’s immoral online behavior was the most lenient, although they did not differ significantly from the bystander group. These findings reflect in-group favoritism and out-group bias in social attribution and moral judgement [23,36,37]. As prior research has shown, the victims often view aggressive behaviors as more unfair and damaging than do the perpetrators, who tend to minimize the harm done [28,29,30,31,54]. Accordingly, when people take the role of the victim, they perceive greater harm done by the perpetrator, and thus they give a harsher moral judgement on the transgression and make less moral observer-licensing compared with when they take the role of the perpetrator or bystander. 

## 6. General Discussion

The current research is the first to investigate moral observer-licensing in the context of cyberspace. When evaluating immoral behaviors in cyberspace, observers condemned the actor less if they knew that the actor had a prior moral history. Although cyberspace has many characteristics such as virtuality and anonymity that differentiate it from the real world, individuals appear to exhibit many similar behavioral patterns online and offline [38,39,45,51], including moral observer-licensing, as revealed in our studies. 

Furthermore, consistent with the moral credits model, although people condoned immoral behaviors in light of the actor’s prior moral history, they still recognized the immorality of the actor’s immoral behaviors [1,14,15,17]. Contrary to what the moral credentials model would predict, prior good deeds reduced observers’ condemnation of a subsequent transgression but did not change their construal of the transgression to non-transgression. It appears that prior moral behaviors do not necessarily cloud people’s moral judgments on subsequent immoral behaviors; rather, they simply make people more forgiving. Importantly, although our findings lend support to the moral credits model in explaining moral observer-licensing in cyberspace, they may be specific to the current context. As researchers have pointed out, moral credentials and moral credits are complementary mechanisms that can both affect moral judgment, and whether licensing occurs via moral credentials or credits is influenced by many contextual factors such as the nature and domain of the transgression [1,49]. Obviously, many important questions remain in terms of how to operationalize and test the differences between the two models, and additional research is needed to examine online moral licensing in light of these theoretical frameworks.

The current research further revealed the influence of role involvement on moral observer-licensing in cyberspace, in particular. When people shared the group identity with the victim, they were less forgiving and more strongly condemned immoral behaviors during moral observer-licensing compared with when they shared the group identity with the perpetrator or the bystander. This finding is in line with the literature showing that people’s perceptions and judgements are biased towards their in-group members [23,31,36,37]. Interestingly, role involvement only influenced the observers’ degree of condemnation, as it did not prevent moral observer-licensing from happening. Regardless of roles, observers condemned the actor less based on the actor’s prior moral behavior. These findings suggest the robustness of moral observer-licensing in moral reasoning. 

Although moral self-licensing has been extensively studied [1,4,47], research on moral observer-licensing is limited. The current studies contribute to the nascent literature. Notably, the moral self-licensing effect is not always found [55,56,57] and the effect sizes exhibit a wide range of variation across studies, with a modest effect size estimate from meta-analyses [47,58]. The current studies on moral observer-licensing showed robust effects, consistent with previous studies on moral observer-licensing [11,49]. The different effect sizes for moral self-licensing versus observer-licensing may reflect different construal levels when people process self- versus other-related information [59], which can further influence the type of judgments people make [60]. It may further reflect the “bias blind spot” where people recognize biased judgments in others while failing to see their own biases [61,62]. It is also important to note that most prior studies on moral licensing focus on participants from WEIRD (Western-Educated-Industrialized-Rich-Democratic) populations [63]. The current studies add to the literature by examining moral observer-licensing in Chinese participants. The strong effects might also reflect the cultural characteristics of the sample, in alignment with prior research with similar samples [11]. This demonstrates the critical importance of examining psychological phenomena beyond WEIRD populations [64,65]. Taken together, future studies should compare moral self-licensing and moral observer-licensing in diverse populations and examine the contributing factors and boundary conditions of each phenomenon. 

Although the current studies yielded original findings about moral observer-licensing in cyberspace and its mechanisms and boundary conditions, there are important limitations. In particular, although conducting the studies in the lab provides experimental control, it also limits the ecological validity of the research. Additional research to examine moral observer-licensing in actual online contexts such as social media will help to corroborate the current findings. Furthermore, future research should examine not only how people perceive an actor’s immoral behaviors in light of the actor’s prior moral history, but also how they subsequently interact with the actor (e.g., whether and how they inflict punishment) [66], which will have important practical implications in real-life settings (e.g., in court). Studies should also examine how people can learn from taking others’ perspectives in making (better) judgments and decisions [62,67]. In addition, it will be fruitful to further examine the type and severity of transgressions in relation to moral observer-licensing online, taking into consideration the social–political issues central in contemporary society. 

In conclusion, the current findings contribute to our understanding of cyber ethics and have important implications for regulating online behavior. They further shed critical light on moral licensing theories in explaining attitudes and behaviors in cyberspace. Although prior good deeds may help individuals gain others’ understanding and forgiveness for subsequent transgressions, it should not be used as an excuse to conduct immoral behaviors. As observers, we need to judge immoral behaviors in cyberspace from a neutral and unbiased perspective. Society should also promote healthy network culture and regulate the cyber environment, reducing and eliminating irrational behaviors such as cyberbullying and cyber-violence. As the Internet continues to exert overarching influences on people’s lives, more research is called for to understand how people think, act, and interact in the cyberspace.

## Figures and Tables

**Table 1:** Frequency distribution of the behaviors.

Type of Behavior		Frequency	Percentage
**Immoral behaviors**			
1	Using or changing others’ accounts online without authorization	32	16.93
2	Internet fraud	30	15.87
3	Spreading rumors or misinformation	27	14.29
4	Producing or spreading computer viruses	27	14.29
5	Prying into others’ private lives	15	7.94
6	Browsing and spreading harmful information	14	7.41
7	Speaking impolitely online	11	5.82
8	Emotional deception online	11	5.82
9	Academic misconduct (e.g., plagiarizing papers online)	7	3.7
10	Producing and spreading information rubbish	6	3.17
11	Flooding the forum with repeated spam messages	4	2.12
12	Sex chat online	3	1.59
13	Internet prank	1	0.53
14	Online copyright piracy	1	0.53
15	Online gambling	0	0
16	Others	0	0
**Moral behaviors**			
1	Supporting social assistance or charitable activities	27	21.43
2	Being honest online	21	16.67
3	Upholding correct guidance of public opinion	17	13.49
4	Protecting others’ intellectual property	17	13.49
5	Providing free consultation or technical support	16	12.7
6	Providing free network management keep the cyber world healthy and safe	10	7.94
7	Disclosure of misbehaviors	9	7.14
8	Speaking politely online	9	7.14
9	Others	0	0

## Data Availability

Data collected and analyzed during the study are available upon reasonable request.

## Supplementary Materials

The following supporting information can be downloaded at: https://www.mdpi.com/article/10.3390/bs12050148/s1, S1 File: Pilot Study data; S2 File: Study 1 data; S3 File: Study 2 data; S4 File: Study 3 data.
